# Research progress of gut microbiota and obesity caused by high-fat diet

**DOI:** 10.3389/fcimb.2023.1139800

**Published:** 2023-03-13

**Authors:** Shuyi Fan, Suyun Chen, Lin Lin

**Affiliations:** ^1^ Scientific Research Department, Brain Hospital of Hunan Province, Second People’s Hospital of Hunan Province, Changsha, Hunan, China; ^2^ Department of Clinical Medicine, Xiamen Medical College, Xiamen, Fujian, China

**Keywords:** gut microbiota, high-fat diet, obesity, probiotics, treatment

## Abstract

Obesity, a chronic metabolic disorder caused by an energy imbalance, has been increasingly prevalent and poses a global health concern. The multifactorial etiology of obesity includes genetics factors, high-fat diet, gut microbiota, and other factors. Among these factors, the implication of gut microbiota in the pathogenesis of obesity has been prominently acknowledged. This study endeavors to investigate the potential contribution of gut microbiota to the development of high-fat diet induced obesity, as well as the current state of probiotic intervention therapy research, in order to provide novel insights for the prevention and management of obesity.

## 1 Introduction

Obesity is a chronic and recurring condition that results from excessive or inappropriate fat accumulation (Obesity: preventing and managing the global epidemic. Report of a WHO consultation, 2000; Bray et al., 2017). The global incidence of obesity among adults has increased by 1.5-fold since 2000, with over 1.9 billion overweight adults in 2016. Children and adolescents have also experienced a rise in the prevalence of obesity, with an increase from 2.9% to 6.8% in the population aged 5 to 19 years (Abarca-Gómez et al., 2017). Obesity has serious implications for health, including an elevated risk of mortality, type 2 diabetes, and cardiovascular disease. The etiology of obesity is multifactorial, with contributing factors including genetics, a high-fat diet (HFD), and gut microbiota. The gut microbiota, which is composed mainly of anaerobic bacteria, facultative anaerobic bacteria, and aerobic bacteria, is a dynamic ecosystem that coevolves with its host (Wu et al., 2022). The gut microbiota plays a crucial role in maintaining the health of the host through vitamin production, nutrient absorption, and the secretion of small molecules involved in immune regulation, angiogenesis, and nerve function (Dominguez-Bello et al., 2019; Robertson et al., 2019). The human gut contains approximately 10^14^ microorganisms (Gill et al., 2006), predominantly composed of Firmicutes and Bacteroidetes species (Bolam and van den Berg, 2018). Different bacterial species occupy distinct sections of the intestine; for instance, Firmicutes often predominate at the top of the gut crypto-villous unit while *Proteus predominates* at the bottom (Sommer and Backhed, 2016). The functional consistency of each bacterial genus is quite high (Costea et al., 2018) and is not affected by the host’s age, sex, BMI, or nationality (Sebastian Domingo and Sanchez Sanchez, 2018).

## 2 Gut microbiota and obesity

### 2.1 Animal studies demonstrate a link between gut microbiota and obesity

The present study shows that the manifestation of obesity and its metabolic dysfunctions were absent in germ-free mice. Notably, the transplantation of cecal or fecal samples from obese mice into germ-free mice resulted in the development of similar symptoms, indicating that the gut microbiota plays a critical role in the pathogenesis of obesity (Ridaura et al., 2013). Furthermore, it was observed that the transfer of gut microbiota could also transmit the obesity phenotype (Kapoor et al., 2021; Romani-Perez et al., 2021). In mice fed the same HFD, some developed obesity and some were resistant to it, and differences in gut microbiota composition may be the most important factor in both outcomes. In addition, intestinal barrier function, intestinal inflammation and neurotrophic factors also play an important role in diet-induced obesity (Zhang et al., 2019b). A growing body of evidence from animal studies suggests a link between diet, gut microbiota and obesity, as well as in humans. But studies have not reached a consistent conclusion on exactly what microbial composition is at work. Moreover, an interesting study found that transfer of the whole microbiota may not reduce diabetes incidence despite a major change in gut microbiota of the non-obese diabetes (NOD) mice model. NOD mouse models can be divided into two colonies (high or low diabetes incidence), transplanting intestinal flora from low-incidence NOD mice to high-incidence NOD mice did not change the incidence of diabetes, but transplantation of *A. muciniphila* to high-incidence NOD mice can promote mucogenesis, increase the expression of antimicrobial peptide Reg3γ, inhibit the growth of rumen contortus, reduce the level of serum endotoxin, reduce the expression of TLR in pancreatic islets, promote regulatory immunity, and delay the development of diabetes (Hanninen et al., 2018). It shows that some single species of bacteria, rather than the entire intestinal flora, may play a major role in inducing or resisting metabolic diseases under certain conditions.

### 2.2 Research on demographics has discovered variations in the distribution of gut microbiota in obese people

As per conventional understanding, the establishment of gut microbiota occurs after birth, while the mother’s uterus remains free of microorganisms. Various factors, such as delivery mode, feeding type, and medication administration (including antibiotics), impact the diversity of gut microbiota, as stated in the literature (Theis et al., 2019; Akagawa et al., 2021). By age 3, the gut microbiota progresses towards a complex and stable state similar to that of adults (Derrien et al., 2019), which then remains mostly consistent throughout adulthood. According to a population-based study, the obese population demonstrates significant differences in gut microbiota composition compared to the general population (Cuevas-Sierra et al., 2019). A few studies propose that the “enterotype of the fertility microbiota” is characterized by a higher abundance of Firmicutes/Bacteroidetes (Kim et al., 2021). Nevertheless, the distribution of this distinct microbiota is still subject to debate due to variation in sample size, individual clinical and anthropometric traits (age, sex, microbiota distribution, and degree of obesity), and microbiota analysis techniques (qPCR, 16S rRNA gene sequencing, and Fluorescence *in situ* hybridization) (Zeng et al., 2019; Assmann et al., 2020).

## 3 A high-fat diet alters gut permeability and gut microbiota in ways associated to obesity

The human gut microbiota is highly responsive to changes in food intake and the physiological state of the digestive system (Turnbaugh et al., 2009; Qin et al., 2020), with alterations observed within a period as short as 24 hours (David et al., 2014). A HFD has been found to significantly reduce the diversity of gut microbiota (Wan et al., 2019), resulting in a decrease in the number of bacteria that are responsible for maintaining the integrity of the gut mucosal barrier and an increase in the number of bacteria that breach it (Monk et al., 2019; Zhang et al., 2019a). This alteration in gut microbiota is characterized by a reduction in the relative abundance of Bacteroides and an increase in the relative abundance of Firmicutes (An et al., 2022). Moreover, the concentration of lipopolysaccharide (LPS) has been found to increase with the number of *Actinomycetes* while the number of *Bifidobacteria* declines as *Vibrio desulfonate* increased. Excess sulfate is converted to hydrogen sulfide, which further compromises the gut barrier and promotes inflammation (Chen et al., 2019). Additionally, the gut barrier is disrupted by *Akkermansia muciniphila (A. muciniphila)*, a member of phylum Verrucomicrobia that degrades mucins and has anti-inflammatory and protective effects on the intestinal mucosal barrier (Hanninen et al., 2018).

### 3.1 Gut permeability is increased by HFD

**Figure 1:**
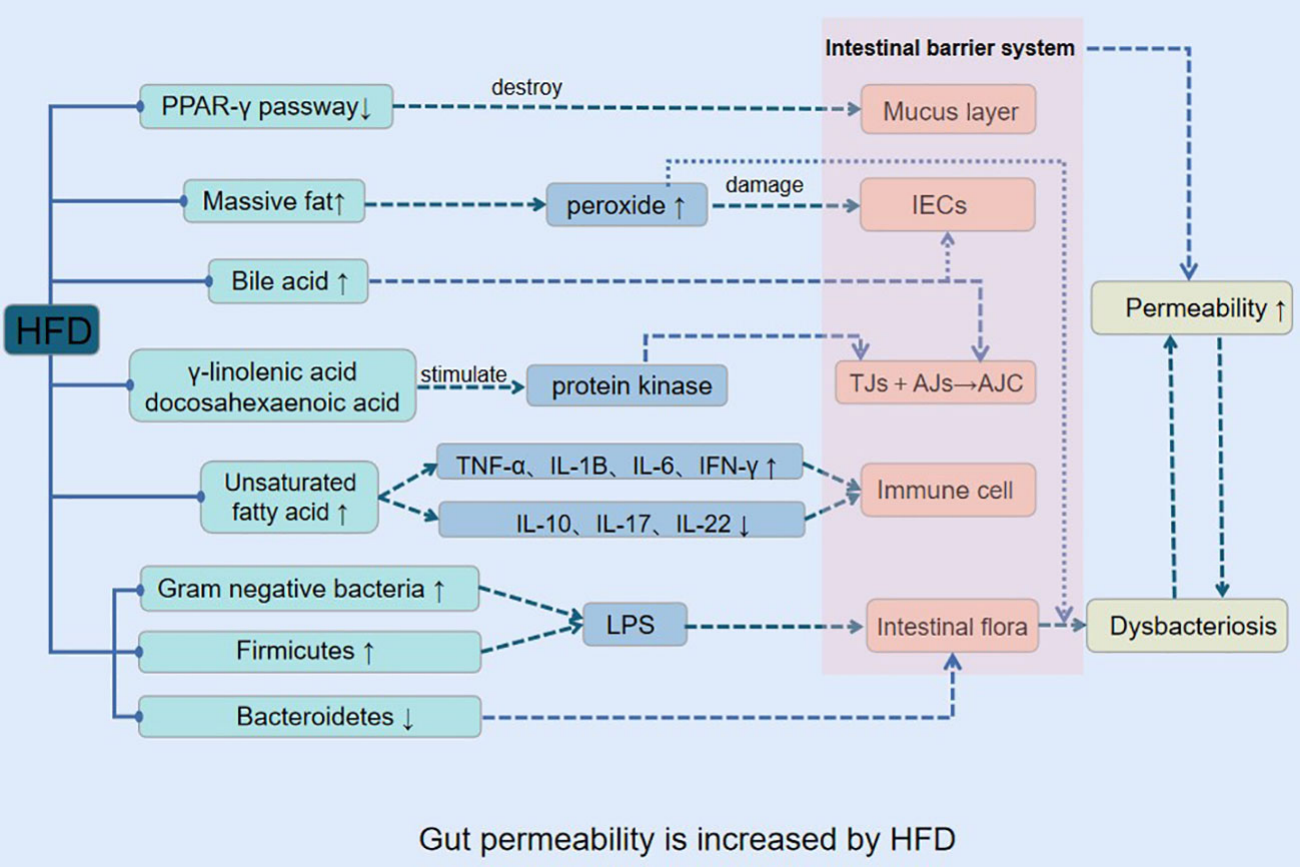
Gut permeability is increased by HFD.

Previous research has provided evidence that a HFD can lead to obesity, inflammation, and enhance gut epithelial cell permeability (Lemons and Liu, 2022). The mechanism through which HFD induces increased gut permeability involves several processes (**Figure 1**).

In the HFD, intestinal epithelial cells in the lower intestine actively ingest a significant amount of fat, which leads to the simultaneous generation of reactive oxygen species (ROS), iron, copper, aldehydes, lipid peroxidation, as well as ATP by the mitochondrial respiratory chain (Spinelli and Haigis, 2018). The ROS generated under the influence of the HFD cause increased gut epithelial cell permeability (Ballard and Towarnicki, 2020), ultimately leading to the destruction of the gut barrier function and the proliferation of harmful bacteria like *Salmonella* and *Escherichia coli* in the gut cavity. Furthermore, the hydrogen sulfide generated by the HFD inhibits the mitochondrial respiratory chain, which makes it easier for pathogenic bacteria to infect more cells (Mottawea et al., 2016). The production of iron, copper, aldehydes, and lipid peroxidation during the digestion and absorption of high dietary fats leads to an increase in oxidative stress in gut tissues, destroying the microbiota’s living environment, resulting in an imbalance of gut microbiota.

The HFD contains a large amount of polyunsaturated fatty acids that are prone to oxidation of their double bonds (Mariamenatu and Abdu, 2021). The free fatty acids generated under the influence of the HFD impact the gut immune system directly (Tanaka et al., 2020) raising the levels of barrier-damaging cytokines such as TNF-α, IL-1β, IL-6, IFN-γ, while decreasing barrier-protective cytokines such as IL-10, IL-17, IL-22, ultimately leading to an increase in gut permeability (Bartoszek et al., 2020; Stoeva et al., 2021). The resulting pathological changes, including low-grade inflammation, decreased expression of antimicrobial peptides, mucus secretion, and expression of tight junction protein, impact multiple system functions and lead to obesity and its metabolic complications (insulin resistance, hyperglycemia, systemic inflammation, and dyslipidemia) (Araújo et al., 2017; Jiang et al., 2020; Kumar et al., 2021).

The gut barrier system comprises mucus layers, gut epithelial cells (IECs), tight junctions (TJS), immune cells, and gut microbiota (Rohr et al., 2020). The apical junctional complex (AJC) is composed of the membrane proteins TJS and adhere junctions (AJS) (Capaldo et al., 2017). The AJC’s integrity is critical for the selective passage of nutrients while obstructing the entry of toxins and antigens, leading to high permeability of the gut. Dietary fat has the potential to directly impact the integrity of the AJC (Netto Candido et al., 2018; Tsukita et al., 2019; Otani and Furuse, 2020). In long-term HFD, gut occlusion zone-1 (ZO-1) and occludin gene expression are decreased, which leads to an increase in gut permeability (Oliveira et al., 2019; Nascimento et al., 2021). The HFD’s abundance of docosahexaenoic acid and γ-linolenic acid triggers protein kinase activation, actin and TJ protein redistribution, and increased gut permeability (Usami et al., 2003). Additionally, part of the eicosapentaenoic acids in HFD can be converted into bioactive metabolites to increase gut permeability (Usami et al., 2001).

Dietary fat consumption and bile acid secretion exhibit a positive correlation (Ocvirk and O'Keefe, 2021), and IECs possess the ability to resist bile acid degradation under normal physiological conditions. However, HFD induces long-term and high-level secretion of bile acids, resulting in the release of numerous hydrophobic bile acids, such as cholic acid and deoxycholic acid (Iwamoto et al., 2021). These bile acids promote occludin protein dephosphorylation, leading to the dissociation of the adhesive junction complex and ultimately causing an increase in gut permeability. In addition, they can cause harm to the gut mucosal barrier and induce oxidative stress and cell apoptosis in IECs (Raimondi et al., 2008; Di Ciaula et al., 2017; Sarathy et al., 2017; Gupta et al., 2020).

Furthermore, HFD inhibits the peroxisome proliferator-activated receptor-γ (PPAR-γ) pathway in mice, disrupting the gut mucus layer, decreasing electrolyte secretion, and impairing mucosal immune defense. However, a week of treatment with a specific PPAR-γ agonist, rosiglitazone, or a return to a normal diet can reverse the increased gut epithelial permeability caused by HFD (Lee et al., 2020), resulting in the disruption of the gut mucus layer, reduced electrolyte secretion, and decreased mucosal immune defense. Following a week of therapy with rosiglitazone, a particular PPAR-agonist, or returning to the usual diet, this increase in gut epithelial permeability was reversed.

### 3.2 Obesity and other related metabolic diseases are mediated by increased gut permeability, which also encourages gut dysbacteriosis

**Figure 2:**
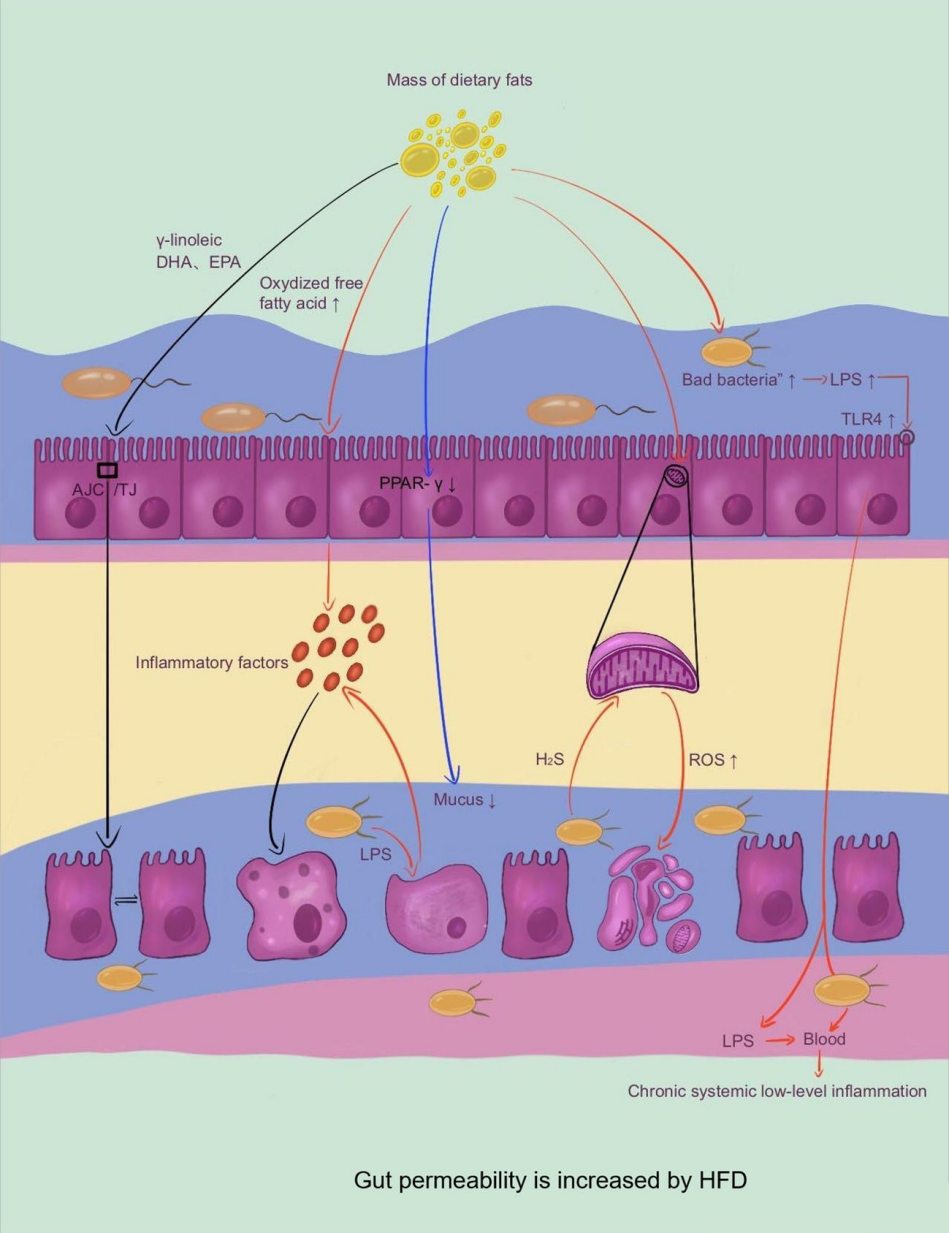
Gut permeability is increased by HFD.

The consumption of a HFD has been observed to enhance the permeability of gut epithelial cells and disrupt the interplay between the local intestine mucosal immune system and the gut microbiota, leading to an imbalance in the microbiota composition. This imbalance is characterized by a rise in the number of gram-negative bacteria, and the resultant LPS produced by these bacteria interact with the CD14/Toll-like receptor 4 (TLR4) complexes of gut epithelial cells, leading to the activation of the innate immune system. This activation causes local and systemic persistent low-level inflammation, which leads to further destruction of the mucous layer and increased permeability of IEC. The heightened permeability of IECs facilitates the entry of gut microbiota metabolites into the bloodstream, resulting in a vicious cycle of inflammation and dysbacteriosis. The ongoing activation of the LPS/TLR4 signal pathway is believed to be a major contributor to the development of obesity and related metabolic disorders (Kasselman et al., 2018; Giordano et al., 2020; Mohammad and Thiemermann, 2020) (**Figure 2**).

## 4 Obesity is a result of gut microbiota’s involvement in the regulation of the human metabolism

### 4.1 Gut microbiota is directly involved in the expression and regulation of host metabolism-related genes

The modulation of host gene promoters related to lipid metabolism, obesity, and inflammatory responses by the dominant Firmicutes within the gut microbiota has been reported through recent investigations (Cuevas-Sierra et al., 2019; Amabebe et al., 2020). However, the examination of gut microbiota-obesity association at a population level presents a significant challenge, given the inadequate sample sizes and inadequate representation of individual subjects in existing gut microbiota studies (Stanislawski et al., 2019). This shortcoming necessitates further research efforts towards resolving these limitations.

### 4.2 Gut microbiota intervenes host glycometabolism through metabolic intermediates

The gut microbiota is responsible for the production of short-chain fatty acids (SCFAs) which impact the host’s ability to absorb and store energy from the diet (Blaak et al., 2020). Production of SCFAs, including acetate, butyrate, and propionate, occurs through fermentation of soluble dietary fiber and resistant starch by gut microbiota (den Besten et al., 2013). The SCFAs bind to the G-protein-coupled receptors GPR41 and GPR43 (Kim et al., 2018; Carretta et al., 2021; Moniri and Farah, 2021), and regulate molecular signaling pathways that indirectly affect gene expression, such as increasing the expression of glucagon-like peptide 1 (GLP-1) and peptide YY (PYY) in the gut (Tanaka et al., 2020). Both GLP-1 and PYY have been found to inhibit appetite (Stubbs et al., 2018), reduce body weight, and improve insulin resistance in obese mice (McNabney and Henagan, 2017; Blanco, 2020). However, in the absence of GPR41 signaling, PYY levels in plasma decrease, causing an increase in gut motility and a decrease in the amount of energy gained from meals (Samuel et al., 2008). Moreover, acetate has been found to positively influence appetite, insulin and ghrelin release, and obesity and its associated complications by influencing the parasympathetic neural system (Hernandez et al., 2019). On the other hand, propionate has been shown to produce insulin resistance and hyperinsulinemia, increases glucagon and fatty acid-binding protein production, activates the sympathetic nervous system, and promotes obesity and metabolic abnormalities (Tirosh et al., 2019). Therefore, further research is needed to explore the relationship between changes in the types and quantity of SCFAs and obesity as it appears that SCFAs act as mediators between diet, gut microbiota, and body physiology.

### 4.3 Gut microbiota interferes with the host lipid metabolism by altering enzyme activity

Bäckhed et al. have proposed potential pathways that contribute to the development of obesity (Backhed et al., 2005). One such pathway involves the gut microbiota promoting the absorption of monosaccharide in the gut, thereby increasing triglyceride synthesis in the liver. Furthermore, gut microbiota has been identified as the primary regulator of lipid metabolism, with both promoting and inhibitory effects. Fasting-induced adipocyte factor (FIAF, also known as PPAR-Angiopoietin Related Protein, which is a cell signal glycoprotein hormone) is known to increase adipocytes’ lipoprotein lipase (LPL) activity and fatty acid accumulation (Backhed et al., 2005). Notably, FIAF is produced by various tissues, including white adipose tissue (WAT), the colon, the liver, the heart, and the skeletal muscle (Baek et al., 2021; Montaigne et al., 2021). Studies have shown that *A. muciniphila* fermentation products, such as SCFAs, promote FIAF expression in gut cells through PPAR-γ (Carvalho and Saad, 2013), inhibit LPL and stimulate WAT lipolysis (Thyagarajan and Foster, 2017). In contrast, *Bacteroides thetaiotaomicron* can stimulate lipogenesis by inhibiting FIAF expression (Backhed et al., 2007). Therefore, FIAF may serve as a gut microbiota modulator, influencing lipid metabolism and contributing to obesity. Additionally, the endogenous cannabinoid system (EC) has been implicated in regulating blood lipid and glucose metabolism, with over-activation posing a significant risk for obesity. Specific gut microbiota, such as *A. muciniphila*, can interfere with fat metabolism *in vivo* by blocking EC-driven lipogenesis, promoting adipocyte proliferation, and increasing fat accumulation in adipocytes (Geurts et al., 2011; Forte et al., 2020; Jansma et al., 2021).

## 5 Probiotics are promising to be a new strategy for treating hfd obesity

Currently, clinical approaches to treating obesity involve reducing caloric intake, increasing exercise consumption, using appetite suppressants, and gastrectomy (Blundell et al., 2017; El Moussaoui et al., 2021; Fanti et al., 2021). Nevertheless, these methods exhibit certain limitations such as limited therapeutic efficacy, drug abuse, and a high incidence of complications (Sarwer et al., 2019; Paccosi et al., 2020; Bray and Ryan, 2021). As a result, innovative treatments are necessary.

**Figure 3:**
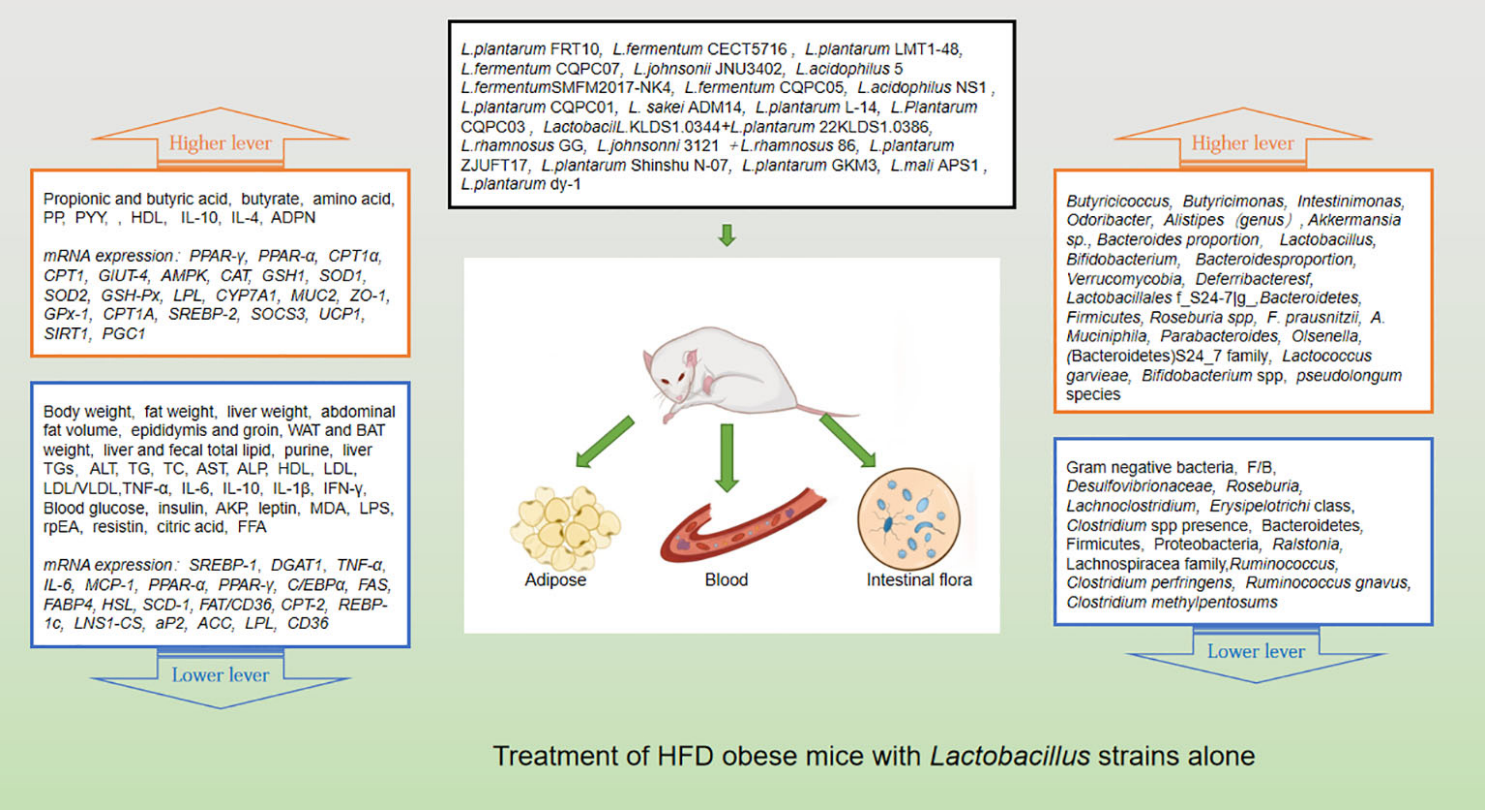
Treatment of HFD obese mice with *Lactobacillus* strains alone.

**Figure 4:**
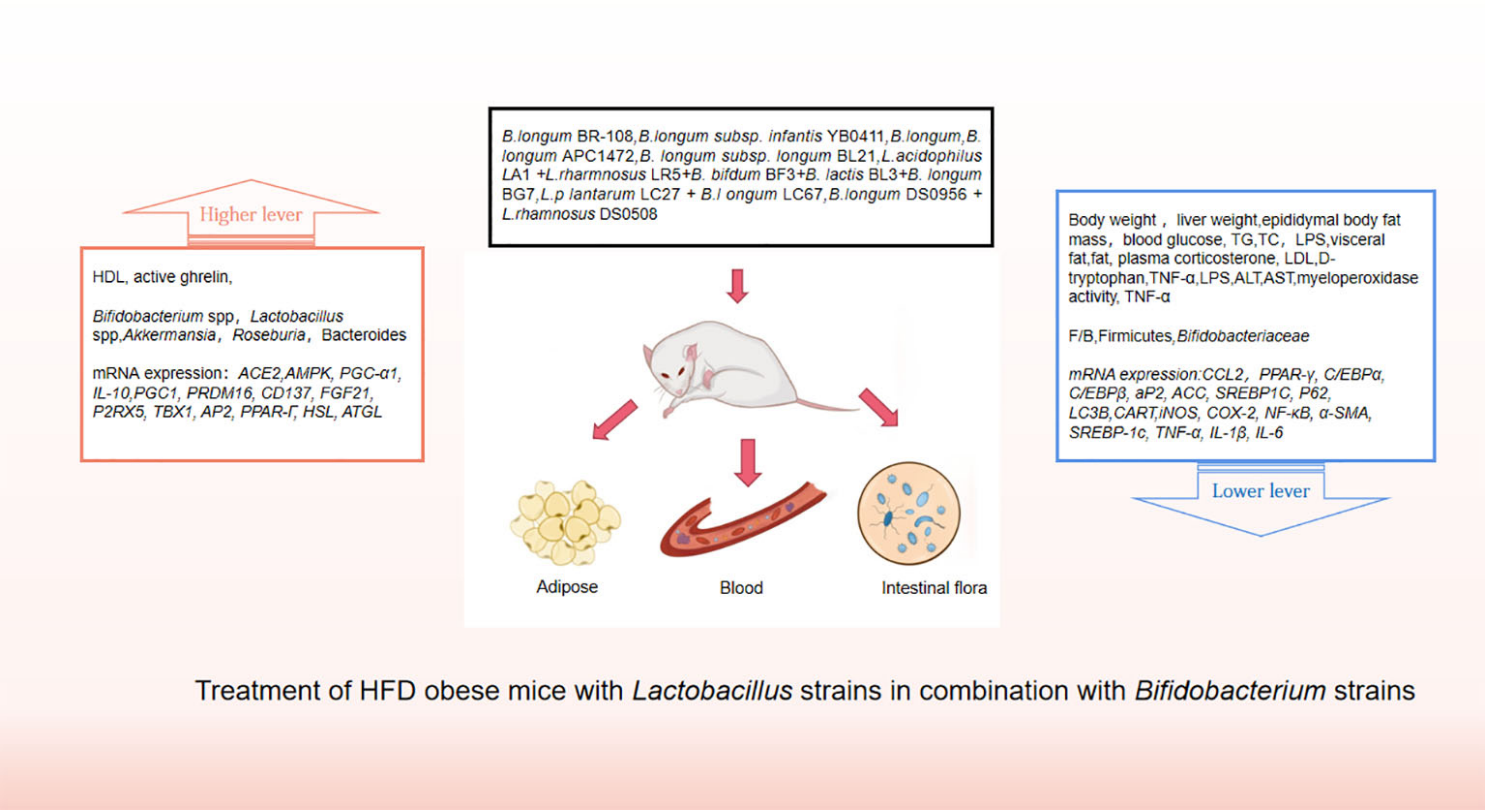
Treatment of HFD obese mice with *Lactobacillus* strains in combination with *Bifidobacterium* strains.

**Figure 5:**
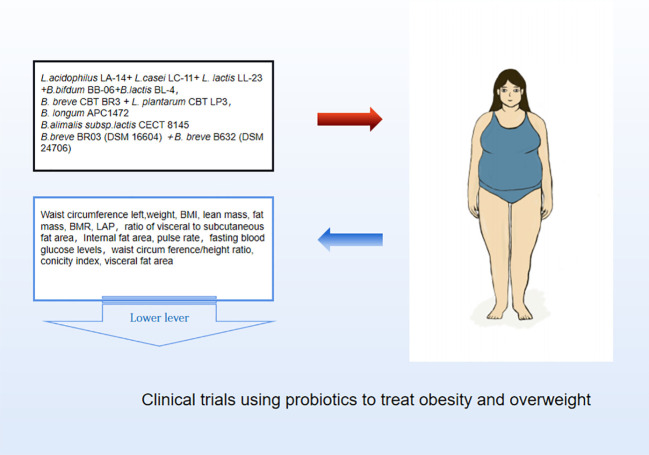
Clinical trials using probiotics to treat obesity and overweight.

Probiotics are living strains that are considered beneficial to the host’s health when consumed in adequate amounts. These microorganisms aid in nutrient digestion and absorption, maintain the digestive system, and improve key metabolic disease risk variables such body mass index, fasting blood glucose, alanine and aspartate transaminase (Jager et al., 2018; Kijmanawat et al., 2019). Utilizing probiotics to regulate gut microbiota has emerged as a promising approach for treating obesity, particularly in cases of HFD obesity (Bianchi et al., 2018; Kong et al., 2019). Numerous animal studies and clinical trials have confirmed the efficacy of probiotics, particularly those from the *Bifidobacterium* and *Lacto bacillus*strains, as well as some members of *Bacillus* and *Propioni bacterium*in treating obesity and overweight by controlling gut microbiota function, bile acid metabolism, and gene expression associated with calorie homeostasis and fat formation (summarized in **Supplementary Tables 1**, **2**). Obese animals treated with multiple *Lactobacillus* strains alone (**Figure 3**) or in combination with *Bifido bacterium*s trains (**Figure 4**) exhibited lower body weight and fat mass, improved dyslipidemia and insulin resistance, and lessened liver damage and chronic low-grade inflammation. Clinical trials using probiotics to treat obesity and overweight have also successfully observed weight loss and improved metabolic markers in subjects, probiotics’s increased presence has negative associations with obesity and diabetes while positively impacting gut health (**Figure 5**). Although data from current human testing studies are limited and urgently need further research and detailed documentation, intestinal bacterial transplantation has emerged in the treatment of HFD obesity and related metabolic issues following successful applications in diseases such as *Clostridium difficile* infection, providing a new option for the prevention and treatment of human HFD obesity.

The gut microbiota of healthy adults and children typically contains 1%-4% of the probiotic *A. muciniphila* (Derrien et al., 2008)*. A. muciniphila* has special survival advantages due to its ability to utilize mucin, the primary growth and metabolic substrate produced by goblet cells in the host gastrogut tissue. Its unique structure enables *A. muciniphila* to modulate gut barrier integrity, enhance gut permeability, and thicken the mucus layer in HFD mice (Chelakkot et al., 2018; Liu et al., 2019). Furthermore, the Type IV pili of *A. muciniphila*are able to directly signal to host immune receptors, regulate the expression of genes involved in fat synthesis and inflammation in the liver, and maintain gut immune system homeostasis (Zhou and Zhang, 2019; Kim et al., 2020; Yang et al., 2020; Xiang et al., 2021)*. A. muciniphila* is also capable of secreting oligosaccharides and SCFA, which act as growth substrates for other beneficial bacteria and promote the abundance of microbiota associated with a reduced risk of obesity (Belzer and de Vos, 2012; Clarke et al., 2014; Keshavarz Azizi Raftar et al., 2021). Long-term supplementation with *A. muciniphila* can increase the thickness of the mucus layer of the gut barrier and attenuate the expression of genes and pathways associated with inflammation (Dao et al., 2016; van der Lugt et al., 2019), thus making it a promising candidate for the treatment of HFD obesity and a potential new generation of probiotics.

## 6 Summary and prospect

Recent studies have shown that there is a distinct distribution of gut bacteria in obese individuals compared to those with a normal weight. This suggests that gut microbiota may play a significant role in the development of obesity and related metabolic disorders, as it is involved in energy metabolism through processes such as acquiring energy from the diet, controlling fat storage, controlling fat creation, and controlling fatty acid oxidation. In light of these findings, new therapeutic approaches such as improving high-fat diet obesity, reducing systemic inflammation, and participating in weight control through targeting gut bacteria have been explored with some success. However, human gut microbiota is a complex research area with various influencing factors, including nutrition, exercise, medications, country, and gender. Some of these variables are beyond our control. Understanding the intricate interaction between billions of distinct bacterial populations, thousands of host cell types, and chemical mediators requires developing well-designed and suitable experimental models. Probiotics have emerged as a safe and effective option for treating HFD-induced obesity in animals, with few adverse effects and good tolerance, making them ideal for long-term administration (Liu et al., 2017), and the combination of *Lactobacillus* and Bifidobacterium has been shown to significantly alter gut microbiota composition and improve insulin sensitivity in HFD mice. In clinical trials, the use of synbiotic bacteria (*Bifidobacterium* and *Lactobacillus*) supplements increased the number of potential probiotics[148], however, it was discovered that the species and quantity of lactic acid bacteria were much higher in obese individuals than in the control group (Armougom et al., 2009), leading to the hypothesis that obese patients may exhibit “resistance” to lactic acid bacteria, which may due to the widespread usage of *Lactobacillus* as a growth stimulant in agriculture. In 2011, MetaHIT team proposed the concept of enterotypes, which divided gut microbiota into three categories: B, P and F. This has potential research and clinical value, but it is controversial. According to different tests, algorithms and analysis methods, different people think that the gut microbiota should be divided into 2, 4 enterotypes or even continuous undivided types. In order to unify the understanding and guide the practice, 29 mainstream microflora scientists in the world jointly proposed a new intestinal type classifier and open comparison database. The new scheme makes full use of and verifies the database such as HMP, comprehensively considers the function, ecology and clinical needs of the flora, and can better indicate the flora types of disease and health status, however, the consensus is significant but still limited, the treatment of obesity still cannot “model” the use of probiotics according to the existing enterotypes classification, and use of personalized probiotics based on precise analysis of each patient’s gut bacteria composition is not yet feasible.

Probiotics therapy may be a novel option for treating HDF-induced obesity, and recent research has shown that using synbiotic supplements and isolating new probiotic strains could increase the potential benefits of probiotic therapy. Nevertheless, it is important to note that a brief course of probiotics may not undo the long-term effects of a physiological disorder, and more research is required to fully understand the role of probiotics in appetite control (Liang et al., 2021).

Although intestinal bacteria transplantation has shown potential for disease prevention and treatment in both animal and human experiments, there are still great controversies over enterotypes, the selection of specific transplant strains and the combination of prebiotics. Since its establishment, microbiology has been limited by axenic culture, but the emergence of mixed culture mode opens up another way for understanding microorganisms and application development, and also has a profound impact on microbial ecology, symbiosis, pathology and other fields. The transition from pure culture to hybrid culture depends on three advances: microfluidic technology, next-generation 3D bioprinting, and single-cell metabolomics. The progress of these technologies is expected to lead to systematic large-scale symbiotic culture studies involving three or more microorganisms in the future. On the basis of in-depth understanding of the correlation between specific enterotypes and metabolic diseases, mixed culture will greatly accelerate the clinical transformation of intestinal bacteria transplantation research. As microbiota science and analytical technology continue to advance, targeted gut microbiota intervention presents potential therapeutic options towards promoting host health in the future.

## Author contributions

SF is the first author, responsible for consulting the literature and forming the first draft, and LL is the corresponding author, responsible for revising the draft. All authors contributed to the article and approved the submitted version.
