# Supplementary material for: Research progress of gut microbiota and obesity caused by high-fat diet
Source: Front Cell Infect Microbiol. 2023 Mar 13;13:1139800. doi: 10.3389/fcimb.2023.1139800 (PMC10040832; doi:10.3389/fcimb.2023.1139800)
Supplement: Supplementary file 1 [file Table_1.docx]

Supplementary Material

Research Progress of GutMicrobiota and Obesity Caused by High-fat Diet

FAN Shuyi^1,2^, CHEN Suyun^2^, LIN Lin^1^*

*** Correspondence:** Lin Lin: [1550770732@qq.com](mailto:1550770732@qq.com)

# Supplementary Tables

Table 1 Treatment of obese / overweight animal with *Lactobacillus* spp. and *Bifidobacterium* spp.

| Treatment | Subjects | Reduced indicators | Elevated indicators | Strain changes | | Reference |
| --- | --- | --- | --- | --- | --- | --- |
|  |  |  |  | Abundance reduce | Abundance increase |  |
| *L.plantarum* FRT10 | Female Kunming mice | Body weight, fat, liver TGs, ALT  mRNA expression: *SREBP-1, DGAT1* | mRNA expression: *PPAR-α, CPT1α* | *Desulfovibrionaceae, Roseburia, Lachnoclostridium* | *Butyricicoccus, Butyricimonas, Intestinimonas, Odoribacter, Alistipes (genus)*  *Lactobacillus, Bifidobacterium, Akkermansia* | (Cai et al., 2020) |
| *L.fermentum* CECT5716 | Male C57BL/6J mice | Body weight, fat, and blood glucose  mRNA expression: *TNF-α, IL-6, MCP-1,PPAR-α* | mRNA expression: *GLUT-4, AMPK* | Erysipelotrichi class, *Clostridium* spp. | *Akkermansia* sp*., Bacteroides* proportion. | (Molina-Tijeras et al., 2021) |
| *L.plantarum* LMT1-48 | Male C57BL/6N mice | Body weight, fat, liver weight, liver TGs, abdominal fat volume  mRNA expression: *PPAR-γ, C/EBPα, FAS, FABP4, HSL, SCD-1, FAT/CD36* |  |  |  | (Choi et al., 2020) |
| *L.fermentum* CQPC07 | C57BL/6J mice | TC, LDL, TG,IL-1β, TNF-α, IL-6, IFN-γ, ALT, AST, ALP  mRNA expression: PPAR-γ | HDL, IL - 10, IL - 4  mRNA expression: *CAT, GSH1, SOD1, SOD2, GSH-Px, CPT1, PPAR-α, LPL, CYP7A1* |  |  | (Wu et al., 2021) |
| *L.johnsonii* JNU3402 | Male C57BL/6J mice | Body weight, liver, epididymis and groin WAT and BAT weight, TG | mRNA expression: *PPAR-γ* |  |  | (Yang et al., 2020) |
| *L.acidophilus* 5 | Male C57BL/6 mice | Body weight, TG, TC, HDL, LDL, TNF-α, IL-6, IL-6, IL-10 | *mRNA expression : MUC2, ZO-1* | Bacteroidetes, Gram negative bacteria | Firmicutes*,* Verrucomycobia *(Akkermansia muciniphila)* | (Ondee et al., 2021) |
| *L.fermentum* SMFM2017-NK4 | C57BL/6J mice | Body weight, TC, TG, ALT, AST  mRNA expression: *FAS, CPT-2* | mRNA expression: *SOD2, CAT, GPx-1* |  |  | (Kim et al., 2021) |
| *L.fermentum* CQPC05 | C57BL/6J mice | ALT, AST, TC, TG, LDL  mRNA expression: *PPAR-γ, C/EBP-α* | HDL  mRNA expression: *LPL, PPAR-α, CYP7A1, CPT1A* |  |  | (Zhu et al., 2019) |
| *L.acidophilus* NS1 | Male C57BL/6J mice | Blood glucose, insulin, TNF-α, leptin  mRNA expression: *SREBP-1c, LNS1-CS* | ADPN  mRNA expression: *PPAR-α, AMPK, SREBP-2* |  |  | (Park et al., 2018) |
| *L.plantarum* CQPC01 | C57BL/6J mice | Body weight, ALT, AST, AKP, TC, TG, LDL, IL-6, IL-1β, TNF-α, IFN-γ  mRNA expression: C/EBP-α, PPAR-γ | HDL  mRNA expression: CYP7A1, CPT1, LPL, CAT, SOD1, SOD2 |  |  | (Gan et al., 2020b) |
| *L. sakei* ADM14 | C57BL/6J mice | Body weight, blood glucose, TC, HDL,  mRNA expression: *FAS, PPAR-γ, C/EBPα, aP2, CD36* | Butyrate | F/B | Bacteroidetes, Deferribacteres | (Won et al., 2020) |
| *L.plantarum* L-14 | C57BL/6J mice | Body weight, TC, HDL  mRNA expression: *PPAR-γ, C/EBPα,* | mRNA expression: *AMPK* |  |  | (Lee et al., 2021b) |
| *L.plantarum*CQPC03 | C57BL/6J mice | Liver TG, TC, LDL, ALT, AST, ALP, MDA, IL-6, IL-1β, TNF-α, IFN-γ | HDL, IL-4, IL-10  mRNA expression: *SOD, GSH-Px, CPT1, LPL, CAT, SOD1* |  |  | (Gan et al., 2020a) |
| *L.lactobacil* KLDS1.0344 + *L.plantarum*22 klds1. 0386 | C57BL/6J mice | TC, TG, LDL, ALT, AST  mRNA expression: *ACC, FAS, PPAR-γ* | HDL  mRNA expression: *CAT, SOD, GSH-PX, GSH* | Firmicutes, F/B | Bacteroidetes | (Li et al., 2020) |
| *L.rhamnosus* GG | Male Balb/C mice | Body weight, fat weight  mRNA expression: *ACC, FAS, SCD* | mRNA expression: *SOCS3* | Proteobacteria, | Bacteroidetes | (Cheng and Liu, 2020) |
| *L.johnsonni* 3121 + their lives hamnosus 86 | Male C57BL/6J mice | Body weight, WAT, TC, LDL/VLDL  mRNA expression: *PPAR-γ, C/EBPα, LPL, CD36* |  | Firmicutes, F/B | *Roseburia* spp*., F. prausnitzii, A.muciniphila* | (Lee et al., 2021a) |
| *L.plantarum* ZJUFT17 | Male C57BL/6J mice | Body weight, blood lipids, LPS, IL-1β, TNF-α | ADPN | *Ralstonia* | *Parabacteroides,Olsenella,Bifidobacteriu* | (Liu et al., 2020) |
| *L.plantarum* Shinshu N-07 | Male C57BL/6J mice | Body weight, epididymal fat, TC, glucose,  mRNA expression: *PPAR-γ* | mRNA expression: *UCP-1* |  | *Lactobacillales*f_S24-7\|g_ | (Yin et al., 2020) |
| *L.plantarum* GKM3 | Male Wistar rats | Body weight, adipose tissue, TC, TG, LDL/HDL, HDL, liver and fecal total lipids, purines | mRNA expression: *SIRT1, PGC1* |  |  | (Hsu et al., 2019) |
| *L.mali* APS1 | Germ-free mice | Body weight, TG, eWAT, rpEA, iWAT, liver weight, liver TG, resistin  mRNA expression: *ACC, FAS, FABP4* | Propionic acid and butyric acid,PP, PYY | Lachnospiracea family*, Ruminococcus*genus*, Clostridium perfringens, Ruminococcus gnavus, Clostridium methylpentosums* | (Bacteroidetes)S24_7 family*, Lactococcus garvieae, Bifdobacterium pseudolongum* species | (Chen et al., 2018) |
| *L.plantarumdy-1* | Male SD rat | Body weight, fat weight, TG, TC, FFA, LDL, citric acid | HDL, amino acid |  |  | (Zhang et al., 2019) |
| *B.longum* BR-108 | Male C57BL/6J mice | Bodyweight, epididymal body fat mass, Blood glucose, TG, TC, LPS  *mRNA expression:ccl2* |  |  | *Bifidobacterium*spp, *Lactobacillus* spp. | (Kikuchi et al., 2018) |
| *B.longum* subsp*. infantis* YB0411 | Male C57BL/6J mice | Body weight, TG  mRNA expression: *PPAR-γ, C/EBPα, C/EBPβ, aP2, ACC, SREBP1C, P62, LC3B* |  |  |  | (Rahman et al., 2021) |
| *B.longum* | Swiss male mice | Body weight, visceral fat, Fasting blood glucose, TC | mRNA expression: *ACE2* |  |  | (Machado et al., 2021) |
| *B. longum* APC1472 | Male C57BL/6J mice | Body weight, fat, plasma corticosterone, fasting blood glucose  mRNA expression: *CART* | HDL, active ghrelin | Firmicutes, *Bifidobacteriaceae* |  | (Schellekens et al., 2021) |
| *B. longum* subsp*. longum* BL21 | Male C57BL/6J mice | Body weight, TC, LDL, D-tryptophan |  | F/B, *Desulfovibrio* | *Akkermansia, Roseburia, Bacteroides* | (Wu et al., 2020) |
| *L.acidophilus* LA1 *+L.rharmnosus* LR5*+B. bifdum* BF3*+B. lactis* BL3*+B. longum* BG7 | Male Sprague Dawley rats | Body weight, liver weight, blood glucose, TG, LDL, TC, LDL | HDL | Firmicutes | Bacteroidetes | (Shin et al., 2018) |
| *L.plantarum* LC27 *+ B.longum* LC67 | Male C57BL/6J mice | Body weight, liver weight, TC, LDL, TNF-α, LPS, ALT, AST, myeloperoxidase activity, TNF-α  mRNA expression: *iNOS, COX-2, NF-κB, α-SMA, SREBP-1c, TNF-α, IL-1β, IL-6* | HDL  mRNA expression: *AMPK, PGC-α1, IL-10* | F/B |  | (In Kim et al., 2019) |
| *B.longum* DS0956 *+ L.rhamnosus* DS0508 | Male C57BL/6J mice | Body weight, fat weight, TCl, HDL, LDL,  mRNA expression: *IL-1β, TNF-α* | mRNA expression: *Pgc1, Prdm16, CD137, Fgf21, P2Rx5, Tbx1, aP2, PPAR-γ, Hsl, Atgl* |  |  | (Hossain et al., 2020) |

Table2 Clinical trials using probiotics to treat obesity and overweight

| Treatment | Duration  (weeks) | Subjects | Outcomes | References |
| --- | --- | --- | --- | --- |
| *L.acidophilus* LA-14*+ L.asei* LC-11*+ L.LL*-23 *+B.bifdum* BB-06*+B.lactis* BL-4*;*  2 x 10^10^ CFU/day | 8 | N = 32,  BMI = 25 to 39.9 kg/m^2^ ,  overweight or obese women | Waist circumference↓, weight↓, BMI↓, lean mass↓, fat mass↓, BMR↓, LAP↓. | (Gomes et al., 2020) |
| *B. breve* CBT BR3 + *L. plantarum* CBT LP3;  1 x 10^10^ CFU/day | 12 | N = 50,  BMI > 25 kg/m^2^,  obesity | waist circumference↓, ratio of visceral to subcutaneous fat area↓，Internal fat area↓, pulse rate↓ | (Song et al., 2020) |
| *B. longum* APC1472;  1 x 10^10^ CFU/day | 12 | N = 124,  BMI = 28-34.9 kg/m^2^,  healthy overweight/obese | not change primary outcomes of BMI or bin-to-hip ratio, fasting blood glucose levels↓ | (Schellekens et al., 2021) |
| *B.alimalis* subsp.*lactis* CECT 8145;  1 x 10^10^ CFU/day | 12 | N = 135,  waist circum ference (WC) ≥ 102 cm for men and ≥ 88 cm for women | Waist circumference↓, waist circum ference/height ratio↓, conicity index↓, visceral fat area↓ | (Pedret et al., 2019) |
| *B.breve* BR03 (DSM 16604) ＋*B. breve* B632 (DSM 24706)  2x10^9^CFU/AFU/day | 8 | N =101，  6-18 years, Tanner stage≥2  obesity and insulin-resistanceyouths | insulin sensitivity↑  weight↓，*Escherichia coli*counts↓ | (Solito et al., 2021) |

Cai, H., Wen, Z., Li, X., Meng, K., and Yang, P. (2020). Lactobacillus plantarum FRT10 alleviated high-fat diet-induced obesity in mice through regulating the PPARalpha signal pathway and gut microbiota. *Appl Microbiol Biotechnol* 104(13)**,** 5959-5972. doi: 10.1007/s00253-020-10620-0.

Chen, Y.T., Yang, N.S., Lin, Y.C., Ho, S.T., Li, K.Y., Lin, J.S., et al. (2018). A combination of Lactobacillus mali APS1 and dieting improved the efficacy of obesity treatment via manipulating gut microbiome in mice. *Sci Rep* 8(1)**,** 6153. doi: 10.1038/s41598-018-23844-y.

Cheng, Y.C., and Liu, J.R. (2020). Effect of Lactobacillus rhamnosus GG on Energy Metabolism, Leptin Resistance, and Gut Microbiota in Mice with Diet-Induced Obesity. *Nutrients* 12(9). doi: 10.3390/nu12092557.

Choi, W.J., Dong, H.J., Jeong, H.U., Ryu, D.W., Song, S.M., Kim, Y.R., et al. (2020). Lactobacillus plantarum LMT1-48 exerts anti-obesity effect in high-fat diet-induced obese mice by regulating expression of lipogenic genes. *Sci Rep* 10(1)**,** 869. doi: 10.1038/s41598-020-57615-5.

Gan, Y., Chen, H., Zhou, X.R., Chu, L.L., Ran, W.T., Tan, F., et al. (2020a). Regulating effect of Lactobacillus plantarum CQPC03 on lipid metabolism in high-fat diet-induced obesity in mice. *J Food Biochem* 44(11)**,** e13495. doi: 10.1111/jfbc.13495.

Gan, Y., Tang, M.W., Tan, F., Zhou, X.R., Fan, L., Xie, Y.X., et al. (2020b). Anti-obesity effect of Lactobacillus plantarum CQPC01 by modulating lipid metabolism in high-fat diet-induced C57BL/6 mice. *J Food Biochem* 44(12)**,** e13491. doi: 10.1111/jfbc.13491.

Gomes, A.C., Hoffmann, C., and Mota, J.F. (2020). Gut microbiota is associated with adiposity markers and probiotics may impact specific genera. *Eur J Nutr* 59(4)**,** 1751-1762. doi: 10.1007/s00394-019-02034-0.

Hossain, M., Park, D.S., Rahman, M.S., Ki, S.J., Lee, Y.R., Imran, K.M., et al. (2020). Bifidobacterium longum DS0956 and Lactobacillus rhamnosus DS0508 culture-supernatant ameliorate obesity by inducing thermogenesis in obese-mice. *Benef Microbes* 11(4)**,** 361-373. doi: 10.3920/BM2019.0179.

Hsu, C.L., Hou, Y.H., Wang, C.S., Lin, S.W., Jhou, B.Y., Chen, C.C., et al. (2019). Antiobesity and Uric Acid-Lowering Effect of Lactobacillus plantarum GKM3 in High-Fat-Diet-Induced Obese Rats. *J Am Coll Nutr* 38(7)**,** 623-632. doi: 10.1080/07315724.2019.1571454.

In Kim, H., Kim, J.K., Kim, J.Y., Jang, S.E., Han, M.J., and Kim, D.H. (2019). Lactobacillus plantarum LC27 and Bifidobacterium longum LC67 simultaneously alleviate high-fat diet-induced colitis, endotoxemia, liver steatosis, and obesity in mice. *Nutr Res* 67**,** 78-89. doi: 10.1016/j.nutres.2019.03.008.

Kikuchi, K., Ben Othman, M., and Sakamoto, K. (2018). Sterilized bifidobacteria suppressed fat accumulation and blood glucose level. *Biochem Biophys Res Commun* 501(4)**,** 1041-1047. doi: 10.1016/j.bbrc.2018.05.105.

Kim, D., Choi, Y., Kim, S., Ha, J., Oh, H., Lee, Y., et al. (2021). Lactobacillus fermentum SMFM2017-NK4 Isolated from Kimchi Can Prevent Obesity by Inhibiting Fat Accumulation. *Foods* 10(4). doi: 10.3390/foods10040772.

Lee, C.S., Park, M.H., Kim, B.K., and Kim, S.H. (2021a). Antiobesity Effect of Novel Probiotic Strains in a Mouse Model of High-Fat Diet-Induced Obesity. *Probiotics Antimicrob Proteins* 13(4)**,** 1054-1067. doi: 10.1007/s12602-021-09752-0.

Lee, J., Park, S., Oh, N., Park, J., Kwon, M., Seo, J., et al. (2021b). Oral intake of Lactobacillus plantarum L-14 extract alleviates TLR2- and AMPK-mediated obesity-associated disorders in high-fat-diet-induced obese C57BL/6J mice. *Cell Prolif* 54(6)**,** e13039. doi: 10.1111/cpr.13039.

Li, H., Liu, F., Lu, J., Shi, J., Guan, J., Yan, F., et al. (2020). Probiotic Mixture of Lactobacillus plantarum Strains Improves Lipid Metabolism and Gut Microbiota Structure in High Fat Diet-Fed Mice. *Front Microbiol* 11**,** 512. doi: 10.3389/fmicb.2020.00512.

Liu, T., Li, Y., Zhao, M., Mo, Q., and Feng, F. (2020). Weight-Reducing Effect of Lactobacillus plantarum ZJUFT17 Isolated from Sourdough Ecosystem. *Nutrients* 12(4). doi: 10.3390/nu12040977.

Machado, A.S., Oliveira, J.R., Lelis, D.F., de Paula, A.M.B., Guimaraes, A.L.S., Andrade, J.M.O., et al. (2021). Oral Probiotic Bifidobacterium Longum Supplementation Improves Metabolic Parameters and Alters the Expression of the Renin-Angiotensin System in Obese Mice Liver. *Biol Res Nurs* 23(1)**,** 100-108. doi: 10.1177/1099800420942942.

Molina-Tijeras, J.A., Diez-Echave, P., Vezza, T., Hidalgo-Garcia, L., Ruiz-Malagon, A.J., Rodriguez-Sojo, M.J., et al. (2021). Lactobacillus fermentum CECT5716 ameliorates high fat diet-induced obesity in mice through modulation of gut microbiota dysbiosis. *Pharmacol Res* 167**,** 105471. doi: 10.1016/j.phrs.2021.105471.

Ondee, T., Pongpirul, K., Visitchanakun, P., Saisorn, W., Kanacharoen, S., Wongsaroj, L., et al. (2021). Lactobacillus acidophilus LA5 improves saturated fat-induced obesity mouse model through the enhanced intestinal Akkermansia muciniphila. *Sci Rep* 11(1)**,** 6367. doi: 10.1038/s41598-021-85449-2.

Park, S.S., Lee, Y.J., Song, S., Kim, B., Kang, H., Oh, S., et al. (2018). Lactobacillus acidophilus NS1 attenuates diet-induced obesity and fatty liver. *J Endocrinol* 237(2)**,** 87-100. doi: 10.1530/JOE-17-0592.

Pedret, A., Valls, R.M., Calderon-Perez, L., Llaurado, E., Companys, J., Pla-Paga, L., et al. (2019). Effects of daily consumption of the probiotic Bifidobacterium animalis subsp. lactis CECT 8145 on anthropometric adiposity biomarkers in abdominally obese subjects: a randomized controlled trial. *Int J Obes (Lond)* 43(9)**,** 1863-1868. doi: 10.1038/s41366-018-0220-0.

Rahman, M.S., Kang, I., Lee, Y., Habib, M.A., Choi, B.J., Kang, J.S., et al. (2021). Bifidobacterium longum subsp. infantis YB0411 Inhibits Adipogenesis in 3T3-L1 Pre-adipocytes and Reduces High-Fat-Diet-Induced Obesity in Mice. *J Agric Food Chem* 69(21)**,** 6032-6042. doi: 10.1021/acs.jafc.1c01440.

Schellekens, H., Torres-Fuentes, C., van de Wouw, M., Long-Smith, C.M., Mitchell, A., Strain, C., et al. (2021). Bifidobacterium longum counters the effects of obesity: Partial successful translation from rodent to human. *EBioMedicine* 63**,** 103176. doi: 10.1016/j.ebiom.2020.103176.

Shin, J.H., Nam, M.H., Lee, H., Lee, J.S., Kim, H., Chung, M.J., et al. (2018). Amelioration of obesity-related characteristics by a probiotic formulation in a high-fat diet-induced obese rat model. *Eur J Nutr* 57(6)**,** 2081-2090. doi: 10.1007/s00394-017-1481-4.

Solito, A., Bozzi Cionci, N., Calgaro, M., Caputo, M., Vannini, L., Hasballa, I., et al. (2021). Supplementation with Bifidobacterium breve BR03 and B632 strains improved insulin sensitivity in children and adolescents with obesity in a cross-over, randomized double-blind placebo-controlled trial. *Clin Nutr* 40(7)**,** 4585-4594. doi: 10.1016/j.clnu.2021.06.002.

Song, E.J., Han, K., Lim, T.J., Lim, S., Chung, M.J., Nam, M.H., et al. (2020). Effect of probiotics on obesity-related markers per enterotype: a double-blind, placebo-controlled, randomized clinical trial. *EPMA J* 11(1)**,** 31-51. doi: 10.1007/s13167-020-00198-y.

Won, S.M., Chen, S., Lee, S.Y., Lee, K.E., Park, K.W., and Yoon, J.H. (2020). Lactobacillus sakei ADM14 Induces Anti-Obesity Effects and Changes in Gut Microbiome in High-Fat Diet-Induced Obese Mice. *Nutrients* 12(12). doi: 10.3390/nu12123703.

Wu, T., Sun, M., Liu, R., Sui, W., Zhang, J., Yin, J., et al. (2020). Bifidobacterium longum subsp. longum Remodeled Roseburia and Phosphatidylserine Levels and Ameliorated Intestinal Disorders and liver Metabolic Abnormalities Induced by High-Fat Diet. *J Agric Food Chem* 68(16)**,** 4632-4640. doi: 10.1021/acs.jafc.0c00717.

Wu, Y., Li, X., Tan, F., Zhou, X., Mu, J., and Zhao, X. (2021). Lactobacillus fermentum CQPC07 attenuates obesity, inflammation and dyslipidemia by modulating the antioxidant capacity and lipid metabolism in high-fat diet induced obese mice. *J Inflamm (Lond)* 18(1)**,** 5. doi: 10.1186/s12950-021-00272-w.

Yang, G., Hong, E., Oh, S., and Kim, E. (2020). Non-Viable Lactobacillus johnsonii JNU3402 Protects against Diet-Induced Obesity. *Foods* 9(10). doi: 10.3390/foods9101494.

Yin, T., Bayanjargal, S., Fang, B., Inaba, C., Mutoh, M., Kawahara, T., et al. (2020). Lactobacillus plantarum Shinshu N-07 isolated from fermented Brassica rapa L. attenuates visceral fat accumulation induced by high-fat diet in mice. *Benef Microbes* 11(7)**,** 655-667. doi: 10.3920/BM2020.0009.

Zhang, J., Xiao, X., Dong, Y., and Zhou, X. (2019). Fermented barley extracts with Lactobacillus plantarum dy-1 changes serum metabolomic profiles in rats with high-fat diet-induced obesity. *Int J Food Sci Nutr* 70(3)**,** 303-310. doi: 10.1080/09637486.2018.1511687.

Zhu, K., Tan, F., Mu, J., Yi, R., Zhou, X., and Zhao, X. (2019). Anti-Obesity Effects of Lactobacillus fermentum CQPC05 Isolated from Sichuan Pickle in High-Fat Diet-Induced Obese Mice through PPAR-alpha Signaling Pathway. *Microorganisms* 7(7). doi: 10.3390/microorganisms7070194.
